# A case report of food-dependent exercise-induced anaphylaxis (FDEIA) treated with omalizumab

**DOI:** 10.3389/falgy.2024.1472320

**Published:** 2024-10-11

**Authors:** Sara Mohamed, Sherin Thalappil, Ramzy Mohamed Ali

**Affiliations:** Allergy and Immunology Division, Department of Medicine, Hamad Medical Corporation, Doha, Qatar

**Keywords:** FDEI, anaphylaxis, omalizumab, wheat, allergy, food dependent exercise induced anaphylaxis

## Abstract

Food-dependent exercise-induced anaphylaxis (FDEIA) is a rare and complex clinical condition in which allergic reactions are triggered by specific foods combined with physical activity, even though the food allergen and exercise are tolerated individually. Wheat is the most common culprit food leading to wheat dependent exercise induced anaphylaxis (WDEIA). Management of FDEIA is challenging due to the unpredictability of attacks and the lack of approved prophylactic medications. This report presents a case of successful symptom control in a young woman with WDEIA through the addition of omalizumab to the treatment regimen. To our knowledge, this is the first reported case of food-dependent exercise-induced anaphylaxis (FDEIA) treated with omalizumab as the primary indication. We also summarize the pathophysiology, diagnosis and treatment of FDEIA. The need for heightened awareness and innovative therapeutic approaches is crucial for those affected by FDEIA.

## Introduction

Food-dependent exercise-induced anaphylaxis (FDEIA) is an uncommon and complex clinical condition where allergic reactions are triggered by the combination of certain foods and physical activity. Individuals can consume the culprit food or engage in exercise independently without any adverse effects, making the condition particularly paradoxical and challenging (1). FDEIA is distinct from typical exercise-induced or food-induced anaphylaxis, where anaphylaxis occurs only after physical exertion alone or intake of the culprit food, respectively (2). Prevalence studies of FDEIA are limited. In Japan, the prevalence among adolescents was estimated to be around 0.017% (3). In the Middle East, no prevalence studies are available, but two cases of FDEIA were reported (4). In a European anaphylaxis registry, wheat-dependent exercise-induced anaphylaxis (FDEIA) to wheat was identified as the most prevalent type of wheat anaphylaxis in adults (6.9% of all 3,646 food reactions). It was more frequent in central Europe (up to 20% of food reactions) than in southern Europe where only a few cases were reported (5). Managing FDEIA poses significant challenges due to variability in individual responses and the potential severity of allergic symptoms, the unpredictable nature of the disease, and the need for strict avoidance of specific food and exercise combinations., as well as the lack of approved preventive/prophylactic medication (6). The current treatment of FDEIA focuses on managing acute symptoms when they occur, maintaining a 4–6 h gap between food intake and exercise, and waiting 1–4 h after exercise (6). Food-dependent exercise-induced anaphylaxis can significantly limit the quality of life, particularly when the culprit food is a key component of the diet, as in Wheat-dependent exercise-induced anaphylaxis (7). This report presents a case of a young woman with FDEIA in whom antihistamines, along with the avoidance of wheat and exercise, were insufficient. The addition of omalizumab successfully controlled her symptoms.

To our knowledge, this is the first case of food-dependent exercise-induced anaphylaxis (FDEIA) treated with omalizumab as the primary indication.

## Case description

A 20-year-old female patient presented to the emergency room in June 2022 with generalized hives, pruritus, vomiting and dizziness. She experienced widespread itching, difficulty breathing, and dizziness while walking with her father at a shopping mall after eating a chicken burger. Her father rushed her to the nearest medical facility, where she was found hypotensive with blood pressure (BP) of 80/40 mmHg and maintained oxygen saturation of 95% on room air. She was treated for anaphylaxis with 0.3 mg of intramuscular (IM) epinephrine, 50 mg of intramuscular diphenhydramine, 200 mg intravenous (IV) hydrocortisone, and two litres of IV normal saline. She responded well to this treatment and was placed under observation for 4 h. Afterwards, she was discharged with prescriptions for oral prednisolone 40 mg and levocetirizine 5 mg, as well as an epinephrine autoinjector for emergency use.

Another episode occurred while she was walking with her mother after eating snacks, including pastries. The patient started to feel generalized itchiness, followed by dizziness and difficulty breathing. The mother called the ambulance. Upon initial assessment, her BP was 94/56 mmHg. She received IM 0.3 mg of epinephrine, IM 50 mg diphenhydramine, and 200 mg hydrocortisone IV. Her vitals improved, and she was observed before being discharged with a 5-day course of oral prednisolone 40 mg, levocetirizine 5 mg, and an epinephrine autoinjector for emergency use. The patient then experienced an additional three episodes a few weeks apart, all occurring while she was engaged in domestic housework after meals. She used her epinephrine autoinjector stock, and improved each time, so she did not visit the hospital. Another episode occurred at her relatives’ house after eating pastries. While walking, she began to experience extensive itching and dizziness. She did not have an EpiPen at that time and was admitted to the health centre emergency and found to be hypotensive with a BP of 99/68 and diffuse skin urticarial rashes. She was treated with IM 0.3 mg epinephrine, IM 50 mg diphenhydramine, and IV 200 mg hydrocortisone. The patient was constantly worried about experiencing an attack while outside the house, leading her to limit her social activities. To ensure safety, her mother and she always carried two epinephrine autoinjectors every time they left the house.

During the patient's initial visit to the clinic, her family and she expressed frustration because they could not identify the trigger for the patient's anaphylaxis. The patient believed that her condition was not related to food allergy because she could eat the same food items without symptoms and experienced no issues when exercising while fasting. None of her episodes were preceded by taking nonsteroidal anti-inflammatory drugs (NSAIDs) or insect stings, and there was no connection to her menstrual cycles. The patient had no history of asthma, rhinitis, or food allergy, although she outgrew childhood atopic dermatitis at the age of 12. Additionally, there was no family history of similar conditions.

### Diagnostic assessment

Basic Laboratory investigations showed normal results, including complete blood count and differential, renal and liver functions, thyroid function, and C-reactive protein. Skin tests for wheat, peanut, tree nuts, fish, shellfish, egg, soybeans, sesame, and legumes and common inhaled allergens, as well as latex, were all negative. Specific IgE (SIgE) for wheat was negative. Her baseline tryptase levels were normal (3.29 mcg/L), and her IgE level was 286 IU/ml. The ImmunoCAP Allergen Component testing (Phadia, Uppsala, Sweden) was positive for tri a 19 (omega-5 gliadin) wheat at 9.65 kUA/L, indicating a high IgE level. Alpha-amylase/trypsin inhibitor and nsLTP were negative. Although provocation tests were not conducted, it is important to note that the patient's detailed history confirmed her ability to tolerate exercise and wheat independently, and so justifying the diagnosis of Food-Dependent Exercise-Induced Anaphylaxis (FDEIA) and the decision to omit these tests.

Due to her severely limiting condition, she was advised to take daily antihistamines and to avoid eating for 4–6 h before and 1–4 h after exercising. Additionally, she was instructed to avoid NSAIDs and/or exercise in hot weather. If she experienced flushing, itching, or hives during exercise, she was instructed to stop immediately and administer epinephrine. It was emphasized that she should carry epinephrine autoinjectors with her all the time and seek immediate medical assistance, even if symptoms improved after using epinephrine for prolonged observation and ongoing management and to ensure no second wave of symptoms emerges after the initial reaction has subsided like in rebound anaphylaxis. Furthermore, she was encouraged to maintain a food diary.

During a subsequent visit, she reported that despite regular antihistamine use, she continued to experience symptoms. Over the course of 12 weeks, she had two more episodes, all of which occurred after consuming a meal containing wheat, as indicated by her food diary. Although she tried removing wheat from her diet, she found it difficult to adhere to such a regimen. Her medication was adjusted to increase fexofenadine to 2 tablets (180 mg) twice a day, but this did not improve the frequency of the attacks. At one point, she collapsed while walking to class after a coffee break (she had biscuits and coffee). Due to the impact on the patient quality of life and inadequate symptom control with antihistamines alone, a comprehensive review for omalizumab use was initiated with a multidisciplinary team (MDT), which included certified three senior consultants specialized in allergy and clinical immunology. Given the off-label use, we ensure the usage of omalizumab is meeting the specific criteria outlined in our local protocol for off-label use. This included a detailed review of their previous treatments, severity of symptoms, and an assessment of potential benefits and risks associated with omalizumab use in this context. Following approval from the MDT, Omalizumab was planned to be administered every four weeks for an initial four months, a duration that can be increased according to the patient response. This aligns with current guidelines for off-label use in similar cases (8). Additionally, the patient received a standard dose of second-generation antihistamines daily throughout the study period to manage baseline symptoms and enhance the efficacy of omalizumab.

treatment with omalizumab, an off-label anti-IgE antibody, was initiated at a dose of 300 mg subcutaneously every 4 weeks, in addition to the antihistamine.

**Figure 1:**
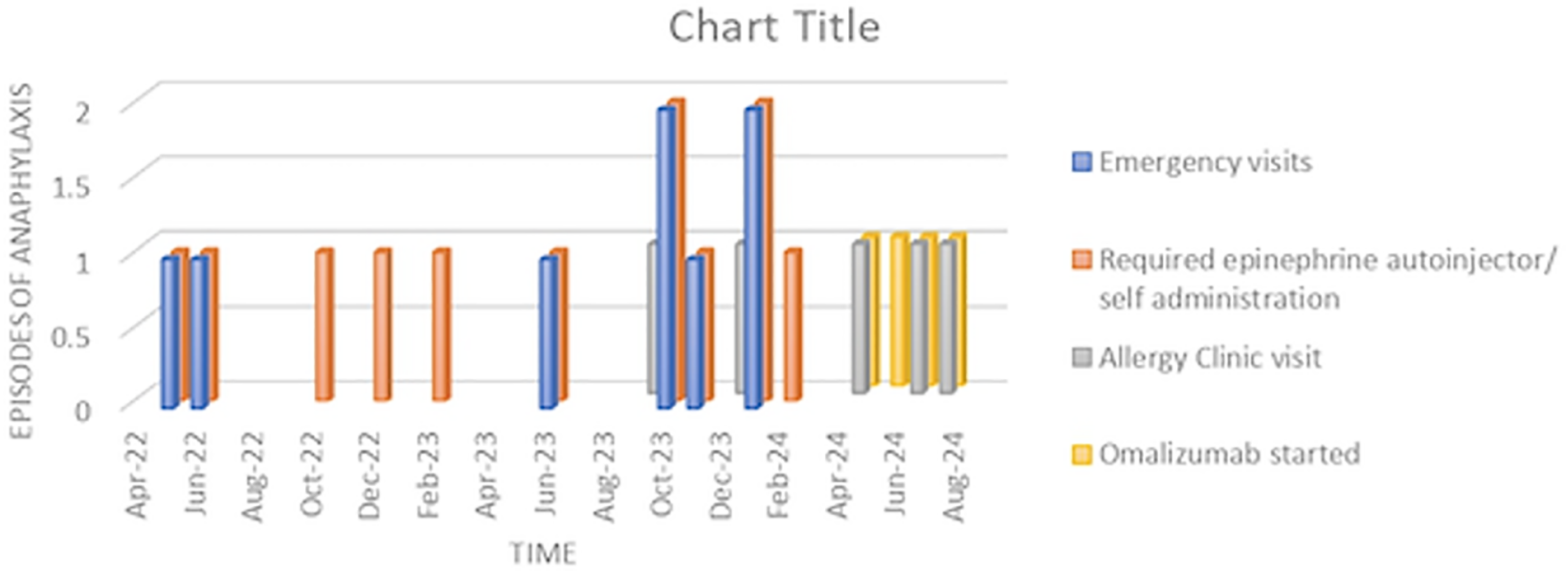
The Episodes of anaphylaxis since diagnosis.

At the three-month follow-up visit after beginning omalizumab injections, the patient was doing well and had not experienced any further anaphylaxis episodes despite continuing to eat wheat-containing foods without spacing out meals and exercise. The severity of her symptoms has significantly reduced (Figure 1). She recently attended a party where she ate cake and pastries and, while dancing, experienced mild itching and dizziness. However, she managed the symptoms by sitting down and taking antihistamines without needing to use her epinephrine autoinjectors. Both the patient and her mother expressed their happiness and satisfaction with the improvement since starting omalizumab.

## Discussion

This case highlights a rare instance of food-dependent exercise-induced anaphylaxis (FDEIA) successfully managed with omalizumab. The patient, despite strict dietary and exercise precautions, continued to experience severe and unpredictable allergic reactions. The introduction of omalizumab, an off-label anti-IgE antibody, significantly improved her condition, reducing both the frequency and severity of her anaphylactic episodes. This outcome underscores the potential of omalizumab as an effective treatment option for FDEIA, particularly in cases unresponsive to conventional antihistamine therapy.

Symptoms of FDEIA typically occur when food is consumed a few minutes to two hours before exercise or rarely up to six hours after exercise (9) and can vary in severity, with anaphylaxis representing the most severe form, which is characterized by urticaria and/or angioedema and associated with symptoms in at least one other organ system, such as respiratory, cardiovascular, or gastrointestinal symptoms (4, 6).

FDEIA often develops in adults, even though cases have been reported in the pediatric population (1). Wheat is the most common food accounting for 60%of cases of FDEIA (10). Omega-5 gliadin, a major protein in wheat, is a contributing factor to over 80% of patients with wheat allergy causing FDEIA (11). In rare cases, high-molecular-weight glutenin subunits (HMW-GS) (12), as well as the wheat lipid transfer proteins (LTP) Tri a 14, are responsible for WDEIA. Anaphylaxis to wheat lipid transfer proteins occurs in severe form and is frequently associated with anaphylaxis to maize and/or rice (13). Other reported triggers of FDEIA include other grains, seafood, peanuts, eggs, milk, Rosaceae fruits and vegetables. In some cases, symptoms may be triggered by any meal followed by exercise, regardless of the food type (14). Additional factors such as alcohol, aspirin, stress, infections, female sex hormones/menstruation (15) and hot and humid weather (16) can act as cofactors in triggering the allergic reaction in FDEIA.

The exact pathophysiology underlying FDEIA remains unclear. Proposed mechanisms include increased basophil activation and mast cell degranulation during aerobic exercise in susceptible patients as well as increased gastric permeability during exercise, which may permit increased entry of intact or incompletely digested allergens into the circulation during exercise but not during rest (17, 18). Another proposed mechanism involves the redistribution of blood away from the viscera to the skin and musculature during exercise may carry food allergens to tissues containing mast cells that are not tolerant to those allergens, resulting in an allergic reaction during exercise but tolerance at rest (19).

Diagnosing FDEIA is challenging and typically relies on a detailed history of the events surrounding the episodes, along with the exclusion of other disorders that could mimic its symptoms. Confirmation of the clinical history can be achieved through either allergy skin prick testing or serum-specific IgE testing. Skin testing can be done with commercially available extract or high-gluten wheat flour. Serum-specific IgE testing for omega-5 gliadin is more clinically useful than for wheat or other wheat allergens, with a sensitivity of around 80% and specificity of more than 95% in WDEIA (20). Measurement of basal serum tryptase levels is also recommended, as elevated baseline levels point out associated mastocytosis or other mast cell disorders. The gold standard for diagnosing FDEIA involves a series of provocation tests, including a food challenge, an exercise challenge, and a combined food-exercise challenge; however, it is not commonly performed in routine medical practice due to the potential risk of inducing a severe anaphylactic reaction during the test, the need for close monitoring and emergency preparedness, and the time and resources required to conduct such a challenge safely (21).

Current management of FDEIA focuses on prompt treatment of acute allergic reactions or anaphylaxis with epinephrine. Patients are educated to avoid the implicated food for 4–6 h before and 1–4 h after exercise or other triggering factors. Prophylactic pharmacotherapy, including antihistamines and cromoglycates, may reduce the frequency and severity of mild symptoms; however, there is currently no evidence that they can prevent anaphylaxis. Data on the use of biologics in FDEIA treatment is limited. Few reports have documented the successful use of dupilumab in a patient with FDEIA (22, 23). While omalizumab use in different types of anaphylaxis has been reported more frequently in the literature. A phase II multicenter single-arm trial of 20 patients demonstrated that long-term omalizumab treatment was safe and effective for adult patients with wheat-dependent exercise-induced anaphylaxis, though it did not achieve desensitization (24). There are also reported cases where omalizumab treatment for chronic spontaneous urticaria has led to improved symptoms of FDEIA (25). Another case detailed a patient with exercise-induced anaphylaxis (EIA) without food triggers who responded to omalizumab with resolution of all EIA episodes. Stopping omalizumab treatment resulted in the recurrence of EIA episodes, which resolved again upon restarting omalizumab, maintaining an EIA-free life for five years (11). Consistently, other reports highlighted the promise of biologics in preventing episodes of exercise-induced anaphylaxis (26, 16).

Omalizumab, marketed as Xolair, is a recombinant humanized immunoglobulin G1 (IgG1) monoclonal anti-IgE antibody that binds to IgE antibodies, preventing their interaction with Fc*ε*RI receptors on mast cells and basophils, thereby inhibiting degranulation and release of mediators of the allergic response. Omalizumab received approval from both the U.S. Food and Drug Administration (FDA) and the European Medicines Agency (EMA) for several indications, including moderate to severe persistent allergic asthma, chronic spontaneous urticaria, chronic rhinosinusitis with nasal polyps and, recently, immunoglobulin E-mediated food allergy for the reduction of allergic reactions (Type I), including reducing the risk of anaphylaxis (27). The approval for food allergies marks a significant step, providing a new way to help manage these conditions. Omalizumab and other anti-IgE therapies increase the threshold of reactivity to foods when given alone, and when combined with oral immunotherapy subsequently, reduce the incidence and severity of adverse events and shorten the time needed for dose escalation (28–31).

This case report illustrates the successful control of FDEIA with omalizumab. Despite the lack of a definitive diagnosis through an exercise provocation test being a limitation, the diagnosis was based on the patient's history and positive specific IgE antibodies to component testing to tri a 19 (omega-5 gliadin) wheat. The effectiveness of omalizumab in FDEIA may be due to its ability to downregulate the high-affinity IgE receptor expression on mast cells and reduce basophil histamine release; however, the exact mechanism of action in FDEIA remains unclear. Further research is needed to better understand the underlying pathophysiology and to offer improved diagnostic and treatment options.

## Conclusion

Food-Dependent Exercise-Induced Anaphylaxis (FDEIA) involves allergic reactions triggered by both food ingestion and physical activity. The exact cause of FDEIA is not yet fully understood. The diagnosis relies on a detailed history. Treatment emphasizes preventive measures, such as allowing a safe interval between consuming food and engaging in exercise, as well as promptly using an epinephrine autoinjector to manage acute reactions. In severe cases where preventive strategies are ineffective or in case of significant limitations in the patient's quality of life, omalizumab can be a beneficial addition, as demonstrated in this case report. It is crucial to raise awareness and develop innovative therapeutic approaches for individuals affected by FDEIA. Exploring novel treatments, such as biologics (including omalizumab) and other innovative therapies, could offer more effective and personalized management options for patients with FDEIA, ultimately improving their quality of life and reducing the risk of severe allergic reactions.

## Data Availability

The raw data supporting the conclusions of this article will be made available by the authors, without undue reservation.
